# A Comprehensive Survey of the Relationship between *Helicobacter pylori* Infection and Atherosclerosis in the Iranian Population: A Systematic Review and Meta-analysis

**DOI:** 10.34172/aim.2022.42

**Published:** 2022-04-01

**Authors:** Masoud Keikha, Mohsen Karbalaei

**Affiliations:** ^1^Antimicrobial Resistance Research Center, Mashhad University of Medical Sciences, Mashhad, Iran; ^2^Department of Microbiology and Virology, Faculty of Medicine, Mashhad University of Medical Sciences, Mashhad, Iran; ^3^Department of Microbiology and Virology, School of Medicine, Jiroft University of Medical Sciences, Jiroft, Iran

**Keywords:** Atherosclerosis, Cardiovascular disease, Helicobacter pylori, Iran

## Abstract

**Background:**

*Helicobacter pylori* is a gram-negative, spiral-shaped, and microaerophilic bacterium that inhabits the human gastric mucosa and is considered to be the most important etiologic agent for gastrointestinal disorders. Recently, however, there is ample evidence to suggest an association between *H. pylori* infection and extragastric complications, particularly atherosclerosis. The aim of this study was to evaluate the rate of *H. pylori* infection and the risk of atherosclerosis in an Iranian population.

**Methods:**

We conducted a comprehensive electronic search on PubMed, Scopus, Google scholar, IranMedex, SID, ISC, and Magiran to find the main published documents related to the relationship between *H. pylori* and atherosclerosis in Iran. A summary odds ratio with 95% confidence interval was used to investigate the potential association between *H. pylori* and atherosclerosis. In addition, the heterogeneity between studies was assessed by the *I *^2^ index and the Cochrane *Q*-test. Publication bias was determined using a funnel plot.

**Results:**

A total of 12 studies met our inclusion criteria and were included in the present study. The results showed that there is a significant positive relationship between infection with this bacterium and the two-fold risk of developing atherosclerosis in the Iranian population (OR: 1.44; 95% CI: 1.07–1.95). However, the heterogeneity was significant and we observed a slight publication bias.

**Conclusion:**

We confirmed a positive relationship between *H. pylori* infection and atherosclerosis in the Iranian population, which is similar to other reports from Western countries. Most likely, *H. pylori* infection can increase the risk of developing atherosclerosis.

## Introduction

 Cardiovascular diseases (CVDs) are the most common cause of death worldwide (50% of global mortality). With an unprecedented increase in mortality over the past two decades, CVD has become one of the major concerns of the global health systems.^1-3^ Atherosclerosis is undoubtedly the most important predisposing factor for cardiovascular disorders with a high prevalence in the developed as well as the developing countries.^2-5^ Atherosclerosis is a chronic disease and several factors are involved in its development.^6^ It should be noted that traditional risk factors such as smoking, obesity, hypertension, hypercholesterolemia, and host genome polymorphisms do not have a significant effect on the pathogenesis of atherosclerosis, and recent studies show that inflammatory diseases play a major role in this regard.^7,8^ Infectious agents are considered as the most important inflammatory triggers in the body, as well as a risk factor for atherosclerosis due to stimulating the inflammatory process and damaging vascular endothelial cells.^9,10^ According to the literature, the most important infectious agents that are associated with atherosclerosis include: *Chlamydia pneumoniae*, *Mycobacterium tuberculosis*,* Helicobacter pylori*, hepatitis C virus,human immunodeficiency virus,Epstein-Barr virus,hepatitis B virus,human T lymphotropic virus type I, andcytomegalovirus.^11-18^ *Helicobacter pylori* is a gram-negative, microaerophilic, and helical bacterium found in the lining of the human stomach and is the etiologic cause of chronic gastritis, gastric ulcer, duodenal ulcer, and gastric cancer.^19,20^ Recent studies have shown that *H. pylori* is also isolated from dental plaques, human saliva, duodenum, feces and atherosclerotic plaques, and is strongly associated with the extragastrointestinal disorders such as idiopathic thrombocytopenic purpura, neurological disorders (stroke events), psychiatric, gynecological, pre-eclampsia, infertility, glaucoma, dermatologic complications, lung cancer, iron deficiency anemia, autoimmune diseases, and atherosclerosis.^21-33^ Unfortunately, the rate of colonization with *H. pylori* is high worldwide, especially in Asia (from 25%–50% in developed countries to 90% in developing countries); the eradication of *H. pylori* infection has decreased in recent years due to problems such as elimination of antibiotics in acidic gastric conditions, bacterial infiltration under the gastric mucosa, and antibiotic resistance.^34-36^ According to studies, the rate of gastric cancer in Asia is higher than Western countries, and many researchers attribute this to the high colonization with *H. pylori* in this geographical area.^37-39^ Moreover, the rate of atherosclerosis in developing countries is higher than developed countries.^4,40,41^ The hypothesis regarding the correlation between *H. pylori* and atherosclerosis was first proposed by Mendall et al in 1994.^42^ Based on extensive studies on the correlation of infection with this bacterium and atherosclerosis, it seems that one of the most important reasons for the increase in atherosclerosis in developing countries could be the high prevalence of colonization with *H. pylori* in these regions (for example, bacteria isolated from atherosclerotic plaques of the patients).^43-45^ However, some sources have ruled out the association between *H. pylori* and atherosclerosis and there is no exact answer to this question, so more studies are needed.^46-48^ The rate of atherosclerosis and cardiovascular disorders is high in Iran and according to the Iranian Society of Atherosclerosis statement, 300 deaths occur due to CVDs in Iran every day. In addition, due to the high rate of colonization with *H. pylori* in Iran (estimated at more than 85%), this country is recognized as one of the most suitable places to investigate the possible link between infection with this bacterium and atherosclerosis. The present study was conducted to investigate the relationship between colonization with this bacterium and susceptibility to atherosclerosis in Iranian patients.

## Materials and Methods

###  Search Strategy

 The present comprehensive meta-analysis was reported based on the Preferred Reporting Items for Systematic Reviews and Meta-Analyses (PRISMA) guideline proposed by Liberati et al.^49^ First, we conducted a fully electronic computer search to retrieve all relevant studies to assess the relationship between colonization with *H. pylori* and atherosclerosis progression. For this purpose, a systematic search was performed using online databases including PubMed, Scopus, Google scholar, as well as national databanks such as IranMedex, SID, ISC and Magiran regardless of publication date and language restrictions by the end of 2019. The search terms were: “*Helicobacter pylori”*, “Iran”, “Atherosclerosis”, “Cardiovascular diseases”, and “Coronary artery disease”.

###  Selection Criteria

 To find eligible studies, the title, introduction, and full text of potentially collected articles were carefully screened independently by the two authors. The inclusion criteria for the selected articles included: I) studies related to *H. pylori* infection in atherosclerotic plaques, II) studies performed in an Iranian population, III) studies published in English or Persian, IV) original studies including cross-sectional, case-control and longitudinal studies, V) articles examining infection by standard methods such as conventional microbiology, PCR, and ELISA, VI) human experiments, VII) studies on clinical specimens, and VIII) full text availability. The exclusion criteria were: 1) *in*-*vitro* studies and non-human investigation, 2) studies containing insufficient results, 3) case reports and review articles, 4) studies evaluating co-infection and other infectious agents other than *H. pylori*, 5) duplicate articles, and 6) studies with abstract only. In addition, the bibliography of the articles was manually searched to prevent loss of related articles.

###  Quality Assessment and Data Extraction

 The Joanna Briggs Institute (JBI) critical appraisal checklist was used to evaluate the quality of the eligible studies. The required data included, first author, publication year, location of studies, frequency of *H. pylori *infection in both case and control groups, diagnostic methods, and references number (Table 1).

**Table 1:** Characteristics of the Iranian Studies About Frequency of H. pylori in CAD Patients

**First Author**	**Year**	**Provinces**	**Type of Study**	**Atherosclerosis Plaque**			**Healthy Individuals**			**Diagnostic Method**	**Ref.**
				**No. HP Positive**	**Total**	**N (%)**	**No. HP Positive**	**Total**	**N (%)**		
Sadeghian et al	2019	Mashhad	Cross-sectional	0	30	0	0	30	0	PCR	^22^
Abibiglou et al	2018	Tabriz	Cross-sectional	1	28	3.57	NA	NA	NA	PCR	^50^
Izadi et al	2012	Tehran	Cross-sectional	56	105	53.33	NA	NA	NA	PCR/serology	^51^
Gharehdaghi et al	2018	Tehran	Case-control	10	90	11.11	0	90	0	PCR	^52^
Nouzari et al	2009	Tehran	Case-control	56	70	80	38	60	65	Serology	^53^
Yazdi et al	2014	Tehran	Cross-sectional	1	90	1.11	NA	NA	NA	Culture	^54^
Pouria et al	2009	Kermanshah	Case-control	8	30	26.66	3	30	10	Serology	^55^
Vafaeimanesh et al	2014	Qom	Case-control	47	62	75.80	10	20	50	Serology	^56^
Sayyah et al	2012	Qazvin	Case-control	32	40	68.08	18	40	45	Serology	^57^
Ansari et al	2010	Urmia	Case-control	49	100	49	56	89	49.5	Serology	^58^
Davoudi et al	2010	Tehran	Case-control	40	69	57.97	48	84	57.1	Serology	^59^
Ashtari et al	2006	Isfahan	Case-control	29	42	69.04	29	43	67.4	Serology	^60^

###  Data Analysis

 The possible relationship between *H. pylori* infection and atherosclerosis was investigated using odds ratio (OR) with 95% confidence intervals (CIs). We used a random-effects model when the heterogeneity was high; heterogeneity was assessed using *I*^2^ statistic and Cochrane *Q* statistic (*I*^2^ > 25% and *P* value < 0.1). The statistical analysis for this study was done using the Comprehensive Meta-Analysis (CMA) software ver. 2.2 (Biostat, Englewood, NJ).^61^ Furthermore, the presence of publication bias was also determined by funnel plot asymmetry, Begg’s *P* value, and Egger’s *P* value tests.

## Results

**Figure 1:**
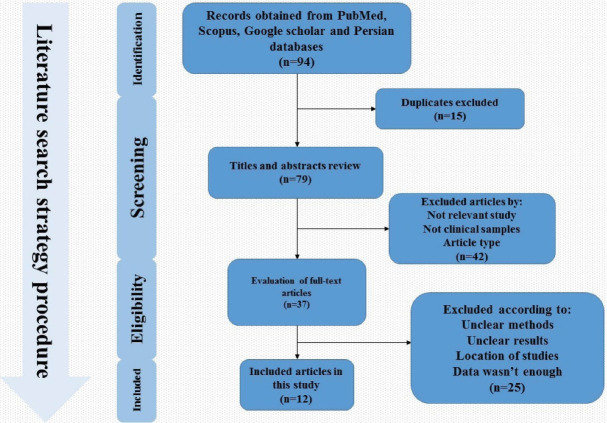
Flowchart of Included Articles According to PRISMA Strategy.

 A total of 94 articles were collected throughout searching in global databases. The titles and abstracts of the studies were screened and the duplicate studies were excluded. Next, 42 studies were excluded in the screening process because they were unrelated to our original idea. Finally, 12articles that met our inclusion criteria were used for the current systematic review and meta-analysis (Figure 1). Of the 12 studies, eight were case-control and four were cross-sectional; all studies were conducted during 2006–2019. Five studies were related to Tehran, and one study had been done in cities such as Tabriz, Mashhad, Kermanshah, Qom, Qazvin, Urmia and Isfahan. In these studies, the information of 1242 participants comprising 756 patients with atherosclerosis and 486 healthy individuals were assessed. Of these, 43.8% were female and 56.2% were male. In addition, the mean age in the case and control groups was 62.7% and 57.4%, respectively.^22,52,59,60^ In four studies, the association between atherosclerosis and *H. pylori *infection was conflicting. In several studies, this relationship was 0–80%.^22,53^ However, none of the selected studies examined the association between eradication of *H. pylori* infection (or *cagA* gene status) and atherosclerosis.

**Figure 2:**
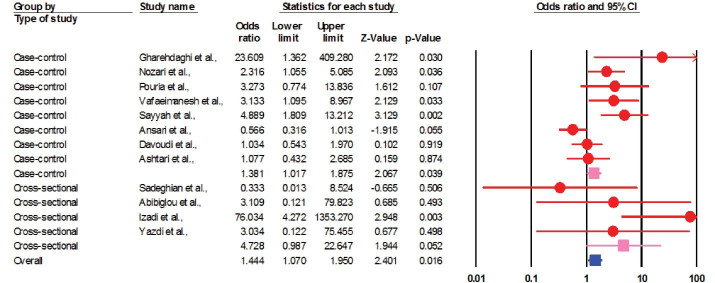
Forest Plot of Pooled ORs of Association between *H. pylori* Infection and Atherosclerosis in the Iranian Population According to Type of Included Studies.

**Figure 3:**
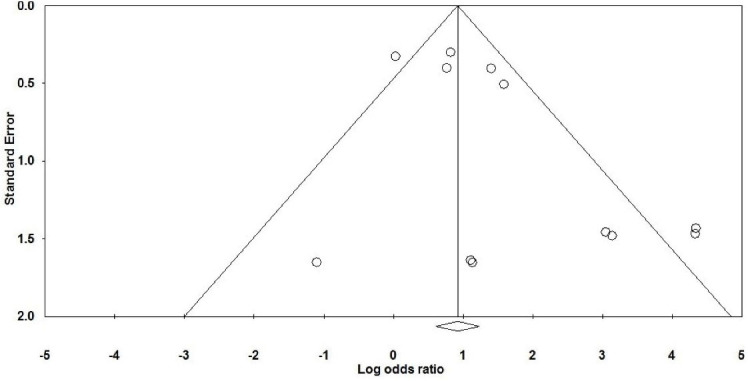
Funnel Plot for Assessment of Publication Bias Which Represent Slight Significant Publication Bias.

 We entered data from 1242 cases in the current meta-analysis; OR is more reliable than relative risk (RR) for predicting risk factors and clinical outcomes, especially in low sample size studies such as the present study.^62,63^ In general, the prevalence of *H. pylori* in atherosclerotic plaques in patients with coronary artery disease (CAD) was estimated at 55.6% (50.3–60.9 with 95% CIs; *P *value: 0.01). On the other hand, the rate of *H. pylori* infection in the control group was measured about 53.7% (49.3–58.1 with 95% CIs; *P *value: 0.01). According to studies, the rate of colonization with this bacterium is high in the population of Iran and we also showed high *H. pylori* infection in both case and control groups, which confirms previous epidemiological studies. Therefore, in case-control studies, the rate of *H. pylori* infection in atherosclerosis cases was significantly higher than normal individuals (*P *value = 0.002). We found a strong positive association between *H. pylori* infection and atherosclerosis in the Iranian population (OR: 1.44; 95% CIs: 1.07–1.95; *P* value: 0.01; *I*^2^: 67.66; *Q*-value: 34.02; *P* value: 0.01; Egger’s *P* value: 0.02; Begg’s *P* value: 0.18) (Figure 2). In addition, due to the significant heterogeneity between studies, we performed subgroup analysis based on the type of study to reduce potential heterogeneity between eligible studies. Based on the results of subgrouping analysis on both case-control (OR: 1.38; 95% CIs: 1.01–1.87; *P* value: 0.03; *I*^2^: 72.50; *Q*-value: 25.45; *P* value: 0.01; Egger’s *P* value: 0.02, Begg’s *P* value: 0.06) and cross-sectional (OR: 4.72; 95% CIs: 0.98–22.64; *P* value: 0.05; *I*^2^: 52.27; *Q*-value: 6.28; *P* value: 0.09; Egger’s *P* value: 0.08; Begg’s *P* value: 0.36) studies, we suggest that *H. pylori* infection can increase the risk of atherosclerosis in the Iranian patients. Also, funnel plot asymmetry showed a slight publication bias in the present study (Figure 3).

## Discussion

**Figure 4:**
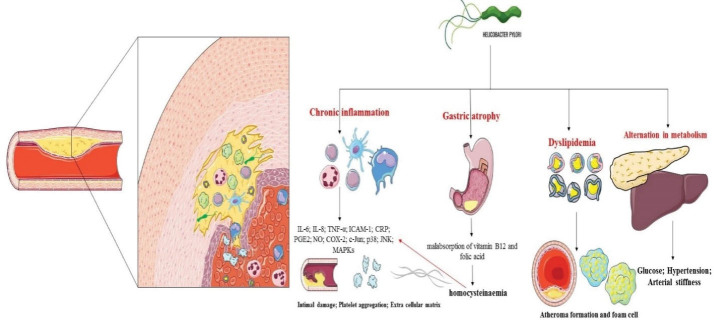
Hypothetic Scheme of *H*. *pylori* Infection and Atherosclerosis (https://smart.servier.com).

 Atherosclerosis is the most common cause of CVDs, especially ischemic heart disease and stroke, and is among the top four causes of death worldwide.^64^ Atherosclerosis is a compound Greek word from *athero* meaning gruel or paste and *sclerosis* meaning hardness. It is a large and medium vascular disease that begins with damage to vascular endothelial cells and changes in blood circulation, followed by the formation of atherosclerotic plaques. The components of plaques include the necrotic cores, calcified regions, lipid particles, smooth muscle cells, endothelial cells, polymorphonuclear cells and foamy cells (alternative macrophages).^64,65^ Vascular endothelium damage (especially intima) and chronic inflammation are the most prominent stimuli for the formation of atherosclerotic plaques.^64-66^ Pathogens such as *C. pneumoniae*, *M. tuberculosis* and *H. pylori*, are among the most important infectious bacteria that can cause chronic inflammation to escape the immune system and appear to play a role in the formation of atherosclerotic plaques.^22^ After decades of early detection, it is now proven that traces of these bacteria are present in coronary, carotid and aortic atherosclerotic plaques.^42,67^ Bahrmand et al showed that *C. pneumoniae* infection is also associated with atherosclerosis; in that study, using PCR, the presence of this bacterium in arterial specimens with severe and mild lesions was 17% and 3%, respectively.^18^ In particular, eradication of *H. pylori* infection reduces C-reactive protein and proinflammatory response and improves endothelial dysfunction, and has a protective effect in the early stages of atherosclerosis.^68-70^ According to this meta-analysis, the rate of colonization with this pathogen is significantly associated with the formation of atherosclerotic plaques in Iranian patients with CAD, and may predispose individuals to cardiovascular disorders (Figure 4).

 Studies have shown that chronic inflammation (such as *H. pylori* infection) during atherosclerosis stimulates the activation of Th1 cells and production of proinflammatory cytokines (IL-1, IL-6, IFN-γ and TNF-α), which in turn leads to the employment of inflammatory cells, especially polymorphonuclear cells. Macrophages also enter into the location in response to MCP1/2, and then induce the inflammatory reactions that lead to the destruction and dysfunction of the endothelial cells.^65-67,71,72^ By stopping the production of stomach acid, this pathogen can lead to atrophy and malabsorption of vitamin B12 and folic acid, resulting in high levels of homocysteine, which stimulates the production of blood nitric oxide by damaging the vessel wall.^73-76^ Recent studies have shown that people infected with *H. pylori* may develop dyslipidemia.^77^ Hoffmeister et al showed that the CAD patients infected with *H. pylori* had higher levels of cholesterol, low-density lipoprotein (LDL), triglyceride, and apolipoprotein-B compared to the CAD patients infected with *C. pneumonia* or cytomegalovirus; high-density lipoprotein (HDL) levels of these patients was also lower.^78^ In contrast, in recovered individuals, serum levels of HDL and apolipoprotein-AI/AII increase, while cholesterol, triglyceride, and LDL levels decrease.^77-79^ Metabolic disorders can also cause atherosclerosis.^80^ Gillum et al showed that there is a significant relationship between *H. pylori* seropositivity and CAD in diabetic patients.^81^ In addition, de Luis showed that CAD and cerebrovascular disorders are high in diabetic patients infected with *H. pylori*.^82^ Polyzos et al found that infection with *H. pylori* is associated with resistance to insulin, and that *H. pylori *infection may lead to atherosclerosis by affecting glucose metabolism, such that glucose resistance improves subsequent to the eradication of the infection while adiponectin (a factor for preventing metabolic disorders) levels increase.^83^ Disorders such as high blood pressure and arterial stiffness play a role in the process of atherosclerosis, and studies have shown that there is a significant relationship between *H. pylori* infection and these complications.^84,85^ Recently, the effect of virulence factors on atherosclerosis has been studied and the previous reports showed a significant relationship between infection with *cagA*-positive strains and carotid plaque.^86-88^ Also, De Bastiani ‎ et al showed that CagA seropositivity is very high in patients with stroke.^89^ In this regard, *cagA*-positive strains have been shown to induce atherosclerosis with effects such as vascular endothelial cell destruction, oxidized LDL modification, and inflammatory stimulation; eradication of these strains stops these injuries and has a protective effect in patients.^90-92^ In addition, it has been now suggested that CagA and heat shock proteins stimulate autoantibody production and endothelial dysfunction, which ultimately leads to atherosclerosis.^93,94^ To assess cardiovascular risk factors, Longo-Mbenza et al followed 205 patients for 10 years in their study and found that *H. pylori* IgG titer was significantly higher in the population of CAD patients.^95^ Following their study on 2029 patients in South Korea, Park et al found that there was a significant relationship between *H. pylori* positivity and CAD patients (OR: 1.23; *P* = 0.049).^96^ Other case-control studies conducted in India, Turkey, and Japan confirmed the results of previous studies.^96-99^ Our study also confirms the relationship between *H. pylori* infection and atherosclerosis in CAD patients. Bacteria isolated from atherosclerotic plaques constitute one of the most important pieces of evidence to confirm the role of this microorganism in atherosclerosis and CAD.^67,94^ So far, extensive studies have been conducted in this area; for example, the rate of *H. pylori* isolation from atherosclerosis in the study by Jha et al was 27.2–33.5%.^97^ In another study in Argentina, the rate of isolated bacterium from carotid plaque was about 83%.^100^ In a cross-sectional study in Turkey, Kilic et al found that the isolation of *H. pylori* from atherosclerotic plaques and non-atherosclerotic vascular wall samples was 48.2% and 19.2%, respectively.^101^ However, *H. pylori* has not been isolated from atherosclerotic plaques in studies conducted in Italy and Poland.^102,103^ Rahmani et al demonstrated in their meta-analysis that there was a significant relationship between the infection with *H. pylori* and myocardial infarction in the Iranian patients (OR: 2.53).^104^ In a meta-analysis of 18 epidemiological studies, Danesh et al found no significant relationship between *H. pylori* infection and coronary heart disease.^105^ In a meta-analysis performed by Wang et al on a 4041 of stroke patients, there was a significant relationship between *H. pylori* infection and ischemic stroke.^23^ In another study by Yu et al, a significant relationship was observed between *H. pylori* infection and CAD in the European (OR: 2.11) and American (OR: 1.43) patients.^106^ However, in another meta-analysis, 13 studies were reviewed and no significant relationship was found between *H. pylori* infection and stroke.^107^ Because recent studies have suggested that infection with strains lacking CagA and VacA may lead to controversial results, the role of virulence factors in CVDs has received more attention.^67,94^ In a study on 684 CAD patients, Mayr et al found that infection with *cagA*-positive strains was significantly associated with atherosclerosis (*P* = 0.08).^108^ In a cross-sectional study on seven case-control studies, Cremonini et al found a significant relationship between stroke and CagA seropositive strains (OR: 1.65).^109^ In addition, Sun et al showed that there was no relationship between *cagA*-positive strains infection and coronary heart disease (OR: 0.8).^110^ Our study had several limitations including (I) small number of studies, (II) in some studies, diagnosis was based on the evaluation of *H. pylori *IgG seropositivity, but this test also yields false positive results after treatment, (III) studies were restricted to geographic regions and this led to controversial results, (IV) the type of CAD was not considered in several studies (e.g. coronary disease, ischemia, stroke, etc.), (V) virulence factors such as CagA and VacAs1m1 were not investigated in all included studies, (IV) heterogeneity was significant, (VII) presence of publication bias for the studies, (VIII) lack of subgroup analysis due to lack of access to raw data. Therefore, the results of the study must be carefully interpreted. Further research is needed with more participants to confirm the validity of the current findings.

 In conclusion, in the present study, we statistically investigated the effect of *H*. *pylori* infection in development of atherosclerosis in the Iranian population. Our study shows that the rate of *H. pylori* infection in cardiovascular patients is higher compared to healthy individuals. We found a strong positive association between *H. pylori* infection and susceptibility to atherosclerosis risk. Our results are consistent with previous meta-analyses in the populations of Western countries. Most likely, *H. pylori* infection can increase the risk of atherosclerosis by inducting proinflammatory response, lymphogenesis, platelet activation, intima media thickness, and endothelial dysfunction. Based on the available findings, *H. pylori* infection can be considered as a risk factor for CVDs, especially atherosclerosis.
